# Synthetic Alfalfa Infusion Odour Attracts Gravid *Culex quinquefasciatus* Under Laboratory Conditions

**DOI:** 10.1007/s10886-024-01528-4

**Published:** 2024-07-13

**Authors:** Betelehem Wondwosen, Elin Isberg, Göran Birgersson, Sharon R. Hill, Rickard Ignell

**Affiliations:** 1 Department of Zoological Sciences, Addis Ababa University, Addis Ababa, Ethiopia; 2 Disease Vector Group, Department of Plant Protection Biology, Swedish University of Agricultural Sciences, Alnarp, Sweden

**Keywords:** Alfalfa infusion, *Culex quinquefasciatus*, Olfaction, Attractants, Stimulants, Volatile organic compounds

## Abstract

Gravid culicine mosquitoes rely on olfactory cues for selecting breeding sites containing organic detritus. While this capacity of the mosquitoes is used for surveillance and control, the current methodology is unwieldy, unreliable and expensive in time and labour. This study evaluated the dose-dependent attraction and oviposition response of gravid *Culex quinquefasciatus* to alfalfa infusions. Through combined chemical and electrophysiological analyses, bioactive volatile organic compounds (VOCs) in the headspace of alfalfa infusions, eliciting attraction, were identified. While phenolic and indolic compounds were the most abundant bioactive VOCs, additional VOCs, including a monoterpene, were required to elicit a significant behavioural response to the synthetic odour blend of alfalfa infusions. Comparative analysis with the commercially available mosquito oviposition pheromone (MOP) was also conducted demonstrating that this standardised synthetic alfalfa infusion odour blend offers a promising lure for targeted surveillance and control of *Culex* mosquitoes, which may contribute to disease prevention and public health protection.

## Introduction

Plant detritus found in breeding sites constitutes a significant portion of the diet for mosquito larvae (Merritt et al. 1992; Yee et al. 2007), and has a noticeable impact on the development and survival of larvae (Asmare et al. 2017; Kesavaraju et al. 2009; Murrell and Juliano 2008; Ye-Ebiyo et al. 2000, 2003), as well as on the size of emerging adults (Kivuyo et al. 2014; Merritt et al. 1992; Ye-Ebiyo et al. 2000, 2003). To evaluate and select breeding sites containing organic detritus, gravid culicine mosquitoes rely on olfactory cues (Afify and Galizia 2015; Khan et al. 2022 and references therein). Behavioural experiments demonstrate that culicine mosquitoes generally display a species-specific response to the odour emanating from detritus from various plant species or infusions thereof, including, but not limited to *Acacia schaffneri*, *Arundinaria gigantea, Cynodon dactylon*, *Medicago sativa, Quercus alba* and *Quercus virginiana* (Afify and Galizia 2015; Hazard et al. 1967; McPhatter and Debboun 2009; Ponnusamy et al. 2009, 2010).

Infusions crafted from plant matter have found an application as lures in gravid traps (Barrera et al. 2014; Chadee et al. 1993; Du and Millar 1999b; Estallo et al. 2011; Mackay et al. 2013; Maciel-de-Freitas et al. 2008; Millar et al. 1992; Mulatier et al. 2022; Musunzaji et al. 2023; Polson et al. 2002; Rawlins et al. 1998; Reiter and Colon 1991; Reiter et al. 1991; Russell and Ritchie 2004; Sant’Ana et al. 2006; Szumlas et al. 1996; Trexler et al. 1998). These lures have aided in the detection and monitoring of arbovirus vectors, and arbovirus testing, during disease outbreaks (Burkett-Cadena and Mullen 2007; Eiras et al. 2014; Johnson et al. 2018; Morrison et al. 2008; Nasci et al. 2002; Polson et al. 2002; Reiter 1986; Ritchie et al. 2014; Seenivasagan et al. 2019; Strickman 1988; Tsai et al. 1989). Due to their affordability and ease of making, plant infusions made from *M. sativa* (alfalfa) are frequently employed as lures in gravid traps for culicine mosquitoes (Allan et al. 2005; Barrera et al. 2014; Bazin and Williams 2018; Chadee and Ritchie 2010; Eiras et al. 2014; Eiras et al. 2021; Mackay et al. 2013; Reisen and Meyer 1990; Ritchie 2001; Snetselaar et al. 2014; Tarter et al. 2019). Alfalfa infusions trigger attraction of gravid *Culex quinquefasciatus* and stimulate egg laying in *Aedes aegypti* under laboratory conditions (Afify and Galizia 2015; Allan et al. 2005; Day 2016; Hazard et al. 1967; Reisen and Meyer 1990). In semi-field and field environments, the adoption of alfalfa infusion stands out as a promising alternative due to its efficacy, cost-effectiveness and specificity in luring gravid culicine mosquitoes, and thus complements existing control strategies (Bazin and Williams 2018; Chadee and Ritchie 2010; Eiras et al. 2014, 2021; Ritchie et al. 2014). The rapid decomposition of plant infusions and the lack of standardization in making these infusions, however, pose a challenge, as the efficiency of the lure may vary significantly between batches and over time, making their use impractical and unreliable for field application (Bazin and Williams 2018; Mullin 2020).

Gravid culicines respond behaviourally to a complex blend of volatile organic compounds (VOCs) emanating from detritus, or infusions thereof, which originate from either the plant material or the associated microbiota (Arbaoui and Chua 2014; Barbosa et al. 2007; Benzon and Apperson 1988; Carrieri et al. 2009; Du and Millar 1999a; Hazard et al. 1967; Huang et al. 2006; Isoei and Millar 1996; Millar et al. 1992; Ponnusamy et al. 2008, 2009; Sant’Ana et al. 2006; Sumba et al. 2004; Trexler et al. 2003). The most common chemical classes of VOCs associated with plant infusions are indolic and phenolic compounds, as well as short- to medium chain alcohols, esters and ketones (Afify and Galizia 2015; Khan et al. 2022). Several of these VOCs have been tested either individually or in blends, for their ability to attract or stimulate gravid mosquitoes to lay eggs, of which indole, 3-methylindole, phenol, 4-ethylphenol, 4-methylphenol and nonanal have received most attention (Afify and Galizia 2015; Allan and Kline 1995; Du and Millar 1999a; Khan et al. 2022; Millar et al. 1992). When compared to blends, individual compounds generally exhibit relatively lower attractiveness or stimulatory effects on egg laying in gravid mosquitoes (Afify and Galizia 2015; Allan and Kline 1995; Baak-Baak et al. 2013; Du and Millar 1999a; Girard et al. 2021; Khan et al. 2022; Mboera et al. 2000a; Millar et al. 1992). An increased understanding of the bioactive VOCs associated with detritus, and how these regulate the attraction of gravid mosquitoes can provide novel tools for surveillance and control of nuisance and disease vectoring mosquitoes.

This study assessed the dose-dependent attraction and oviposition of gravid *Cx. quinquefasciatus* in response to alfalfa infusions, and aimed to identify a synthetic blend of bioactive VOCs eliciting attraction, employing a combination of behavioural and electrophysiological bioassays. The results of this study may pave the way for the development of a novel lure that can be used in a targeted and environmentally sustainable strategy for mosquito surveillance and control, which ultimately could contribute to disease prevention and public health protection.

## Materials and Methods

### Mosquito Rearing

*Culex quinquefasciatus* (Thai) were reared under controlled laboratory conditions at 27 ± 2 ºC, with a relative humidity of 70 ± 2% and a 12 h:12 h light:dark (L:D) photoperiod. The larvae were reared in plastic trays (20 cm × 30 cm × 10 cm), half filled with distilled water, and fed with fish food (Best Friend Flakes Complete, Best Friend, Solna, Sweden or Supervit Tablets B, Tropical Tadeusz Ogrodnik, Chorzów, Poland). The pupae were collected and transferred to 30 ml containers, which were placed in BugDorm-1 cages (30 cm × 30 cm × 30 cm; Mega View Science, Taichung, Taiwan). Upon emergence, the adult mosquitoes of both sexes were provided ad libitum access to a 10% sucrose solution through a filter paper wick. For colony maintenance and bioassays, 4–5 days post-emergence (dpe) adults were allowed to feed on sheep blood (Håtunalab AB, Bro, Sweden) from a membrane feeder (Hemotek, Discovery Workshops, Accrington, UK) for 1 h. Gravid mosquitoes, 3 d post-blood meal (pbm) were used for the experiments.

### Preparation of Alfalfa Infusions

The alfalfa (*M. sativa*) infusions were made following the methods described by Reiter (1983) and Millar et al. (1992). For the preparation, 225 g of alfalfa hay (Zoogiganten, Lomma, Sweden), 10 g of brewer's yeast, and 10 g of lactalbumen hydrolysate (Sigma-Aldrich Chemie GmbH, Steinheim, Germany) were added to 40 l of tap water, in a closed container, at room temperature, and allowed to sit under anaerobic conditions for 7 days. Thereafter, the infusion was filtered using a sieve and filter paper into 1 l glass bottles. For behavioural assays, the stock infusion was diluted with distilled water to obtain 0.001%, 0.01%, 0.1%, 1% and 10% solutions. For each round of bioassay, a new batch of infusion was prepared to ensure a consistent infusion for the experiments.

### Oviposition Bioassay

To assess the attraction and preference of gravid *Cx. quinquefasciatus* for egg laying in response to the alfalfa infusion, a two-choice oviposition assay, in Bugdorm-1 cages, was used. In each cage, two polypropylene cups (30 ml), filled with either distilled water (10 ml) or an equivalent volume of diluted infusion, were placed in opposite corners of the cage. To mitigate bias, the position of the cups was alternated between experiments. Ten gravid females (3 d pbm) were introduced into the cages at Zeitgeber time (ZT) 10, and then allowed access to the oviposition cups overnight until ZT 2. Thirty replicates were conducted for each treatment.

### Headspace Collection of Alfalfa Infusions

To collect headspace volatiles, a charcoal-filtered continuous airstream (1 l min^−1^) was passed over 0.1 l of a 7-day alfalfa infusion in a sterile glass bottle (1 l) using a diaphragm vacuum pump (KNF Neuberger, Freiburg, Germany) for 3 h. Aeration columns, consisting of Teflon™ tubing (6 cm × 3 mm internal diameter), holding 35 mg of Super Q adsorbent (80/100 mesh; Alltech, Deerfield, IL, USA) between polypropylene wool plugs and Teflon™ stoppers, were used to adsorb the volatile compounds from the headspace. Prior to use, the columns were rinsed with 1 ml of re-distilled *n*-hexane (LabScan, Malmö, Sweden). The adsorbed volatiles from four replicates were subsequently eluted with 300 μl of re-distilled *n*-hexane and pooled for combined gas chromatography and electroantennographic detection (GC-EAD) analysis.

### Electrophysiology

The GC-EAD analysis was conducted using an Agilent 6890N GC (Agilent Technologies, Santa Clara, USA). The GC was equipped with an HP-5MS fused silica column (30 m × 0.25 mm × 0.25 µm; Agilent Technologies), using hydrogen as the carrier gas at an average linear flow of 45 cm s^−1^. Aliquots of the extract (2 µl) were injected in splitless mode (30 s, injector temperature 225 °C). The column temperature was set at 35 °C (3 min hold) and then increased to 290 °C at 10 °C min^−1^ (10 min hold). Nitrogen (4 psi) was added to the effluent from the GC column, and then split 1:1 in a Gerstel 3D/2 low dead volume four-way cross (Gerstel, Mülheim, Germany) directed towards the flame ionization detector and the EAD. The GC effluent for the EAD passed through a Gerstel ODP-2 transfer line (Gerstel), with the temperature tracing that of the GC, into a glass tube (30 cm, 8 mm inner diameter). The effluent from the transfer line was mixed with charcoal-filtered, humidified air (1.5 l min^−1^) and passed over the antenna, placed 0.5 cm from the opening of the glass tube.

For the EAD analysis, the head, including the antennae, was carefully removed. Two glass electrodes, a reference, and a recording electrode, were filled with Beadle-Ephrussi Ringer solution. The reference electrode was inserted into the foramen of the head, whereas the recording electrode was placed over the trimmed tip segment of an antenna. To amplify the signals, the recording electrode was connected to a pre-amplifier (10 ×) and then to a high impedance DC amplifier interface box (IDAC-2; Syntech, Kirchgarten, Germany). At least three consistent recordings were performed from different individual mosquitoes. The recorded antennal responses to the samples were collected and analysed using GC-EAD 2011 software (version 1.2.3, Syntech).

### Chemical Analysis

The extract used for the GC-EAD analysis was subjected to combined GC and mass spectrometry (MS) analysis, using two different types of fused silica capillary columns (60 m × 0.25 mm, 0.25 μm film thickness), DB-Wax (7890B GC and 5977A MS; Agilent Technologies) and HP-5MS UI (6890 GC and 5975 MS; Agilent Technologies). Helium was employed as the carrier gas with an average linear flow rate of 35 cm s^−1^. The column oven program used was consistent with that of the GC-EAD analyses. The volatile compounds detected by the antennal recordings during the GC-EAD analysis were identified based on retention times (Kovats indices) and mass spectra. The mass spectral data were compared with standard library data (NIST05, Agilent Technologies) to aid in the identification process. In addition, the chirality of *α*-pinene and linalool was identified using an HP 6890 GC 5973 MS instrument (Hewlett-Packard, Palo Alto, USA), equipped with a J&W Cyclodex-B column (60 m × 0.25 mm × 0.25 µm; Agilent Technologies), with helium as the carrier gas (32 cm s^−1^). The GC oven program was optimized for peak separation using synthetic standards. A 2 µl aliquot of the alfalfa headspace sample was injected onto the GC–MS, with co-injected synthetic standards aiding peak identification. In addition, antennal responses to binary blends of the chiral compounds, after installing the Cyclodex-B column into the GC-EAD system described above, allowed for validation of the bioactive VOC enantiomer. To quantify the relative abundance of the bioactive compounds, an internal standard of 100 ng of heptyl acetate (99.8%; Aldrich) was added to the headspace extract.

The identified compounds were validated by comparison with authentic standards: methyl isovalerate (Cas no. 556–24-1; Sigma-Aldrich, 98%), 1-heptanol (Cas no. 111-70-6; Sigma-Aldrich, 98%), (±)-*α*-pinene (Cas no. 80-56-8; Aldrich, 97%), (1*R*)-(+)-*α*-pinene (CAS no. 7785-70-8; Sigma, 98%), (1*S*)-(-)-*α*-pinene (Cas no. 7785-26-4; Aldrich, 99%), phenol (Cas no. 108–95-2; Aldrich, 99%), (±)-linalool (Cas no. 78-70-6; Sigma-Aldrich, 99%), (1*S*)-(-)-linalool (Cas no. 126-91-0; Sigma-Aldrich, 98%), 4-ethylphenol (Cas no. 123-07-9; Aldrich, 99%), 4-ethylguaiacol (Cas no. 2785-89-9; Sigma-Aldrich, 98%), indole (Cas no. 120-72-9; Aldrich, 99%) and undecanone (Cas no. 112-12-9; Aldrich, 90%).

### Behavioural Assay with Synthetic Blends

To assess the behavioural attraction of gravid *Cx. quinquefasciatus* to synthetic blends of the VOCs identified from the headspace of alfalfa infusions, a straight tube olfactometer was used (Majeed et al. 2017). The olfactometer (80 cm × 10 cm i.d.) was illuminated from above by red light (40 lx). Charcoal-filtered and humidified air (25 ± 2 °C, 65 ± 2% RH) passed through the bioassay at 30 cm s^−1^. Experiments were conducted during the first two hours of scotophase, which is the peak time for oviposition of *Cx. quinquefasciatus* (Beehler et al. 1993).

A stock solution of the synthetic blend, consisting of the nine electrophysiologically active VOCs at the average detected ratio within the four pooled headspace collections, was prepared. The blend was diluted in pentane (Merck), and released by diffusion from a wick dispenser, with release rates between 1.5 to 450 ng min^−1^ of *α*-pinene. Pentane was used as the control. In addition, subtractive synthetic blends, in which an individual VOC was removed from the full blend, were tested. The wick dispenser was made out of a 1.5 ml glass vial with a hole in the centre of the cap through which a cotton wick encased in Teflon protruded into the air (Karlsson et al. 2013). The wick dispenser allows for the release of all compounds in constant ratio throughout the experiment. The wick dispenser was placed inside a glass wash bottle (250 ml; Lenz Laborglas, Wertheim, Germany). Humidified and charcoal-filtered air (0.3 l min^−1^) was passed through the wash bottle and delivered via low-density polyethylene tubing at the upwind end of the olfactometer. For comparison, the mosquito oviposition pheromone (MOP), 6-acetoxy-5-hexadecanolide (Cas no. 81792-36-1; Bedoukian. Research Inc., Danbury, Connecticut USA) was tested in a dose-dependent manner. The MOP was diluted in *n*-hexane (LabScan) and pipetted onto a 1 cm^2^ filter paper (55 mm; Munktell Filter AB, Ahlstrom-Munksjö AB, Helsinki, Finland), and then suspended from a steal wire at the upwind end of the olfactometer. A filter paper with *n*-hexane only was used as a control. The filter paper was replaced after each replicate.

Two hours prior to the experiments, gravid mosquitoes (4 d pbm), in groups of five, were collected and transferred to release cages. At the time of the experiment, a release cage was placed downwind of the assay and the mosquitoes were allowed 30 s to acclimatize. After 30 s, the butterfly valve of the cage was opened and after two minutes the number of females reaching the upwind end of the olfactometer was recorded, and the proportion of mosquitoes reaching the upwind side of the assay was calculated. Ten replicates were performed for each of the treatments.

### Statistical Analyses

For the oviposition experiments, using alfalfa infusions, an oviposition attraction index (OAI) was calculated as (T − C)/(T + C), in which T represents the number of egg rafts laid associated with the test, and C represents the number of egg rafts laid on the controls. A response index was computed as (S)/(R + N) to analyse data from the attraction assay in the straight tube olfactometer, in which S represents the number of mosquitoes responding to the test odour, R denotes the number of mosquitoes in the release cage and N indicates no response to the test odour. The behavioural responses of gravid *Cx. quinquefasciatus* in the oviposition bioassay and the straight tube olfactometer were evaluated using a binary logistic regression analysis in SPSS Statistics for Windows (Version 20, IBM Corp, Armonk, USA), in which the dependent variable was weighted by the number of eggs laid in the oviposition assay and the number of responding mosquitoes in the attraction assay.

## Results

### Oviposition Preference of *Culex quinquefasciatus* to Alfalfa Infusion

**Fig. 1:**
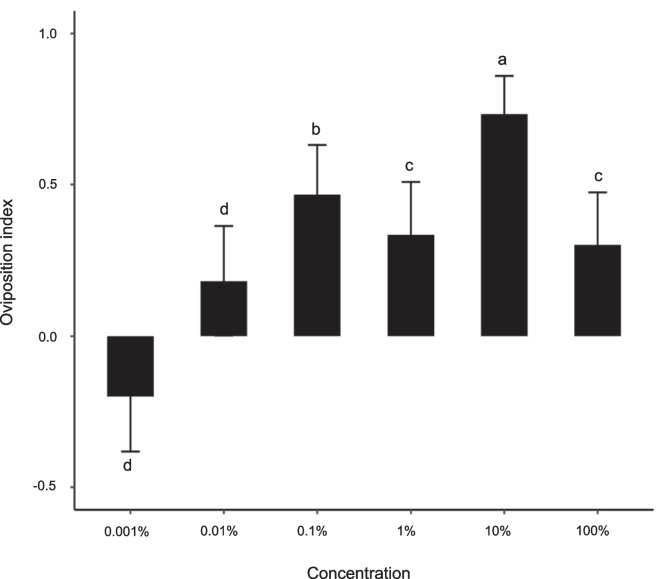
Dose-dependent oviposition response of *Culex quinquefasciatus* to alfalfa infusions. A binary logistic regression was used for statistical analysis. The different letters indicate significant difference in a pairwise comparison. Error bars denote the standard error of the mean. Ten replicates of five mosquitoes each were used in each behavioural experiment

Gravid *Cx. quinquefasciatus* exhibited a dose-dependent attraction and oviposition response to the alfalfa infusion. Females preferred to lay significantly more eggs in the treated versus the control oviposition cups (χ^2^ = 5.61, 95% CI 17.10–35.56; *P* = 0.0001; Fig. 1).

### Antennally Bioactive Compounds in Alfalfa Infusion

**Fig. 2:**
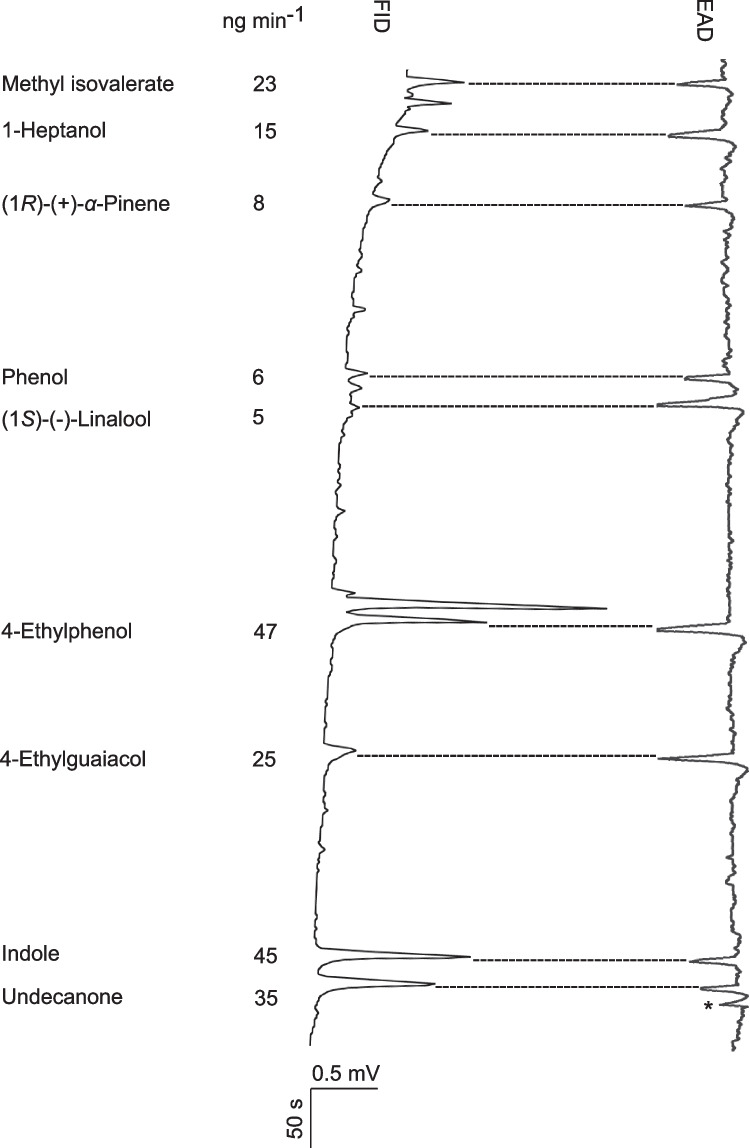
Antennal response of gravid *Culex quinquefasciatus* to alfalfa infusion volatile organic compounds. Combined gas chromatography and electroantennographic detection (EAD), as well as combined gas chromatography and mass spectrometry analyses, were used to identify bioactive volatile organic compounds in the headspace of alfalfa infusions. The electroantennographic detection (EAD) trace illustrates the antennal response (mV) as the bioactive compounds elute from the gas chromatograph column. FID: the flame ionization detector signal of the gas chromatograph. The identity and release rate of the bioactive compounds are shown on the left. The asterisk indicates a response rarely found of the antenna to an unidentified compound

The GC-EAD and GC–MS analyses identified nine bioactive VOCs detected by the antenna of gravid *Cx. quinquefasciatus*: methyl isovalerate, 1-heptanol, (1*R*)-(+)-*α*-pinene, phenol, (1*S*)-(-)-linalool, 4-ethylphenol, 4-ethylguaiacol, indole and undecanone (Fig. 2). The total release rate of these VOCs was approximately 209 ng min^−1^, with 4-ethylphenol and indole as the most abundant VOCs (Fig. 2).

### Attraction to a Synthetic Alfalfa Infusion Odour Blend and MOP

**Fig. 3:**
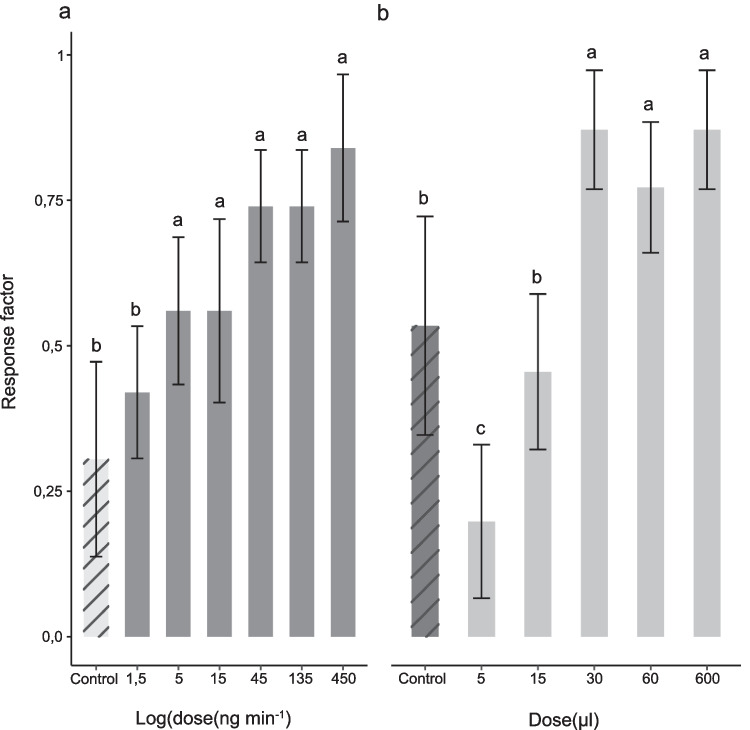
Dose-dependent attraction of gravid *Culex quinquefasciatus* to (**A**) a synthetic odour blend of alfalfa infusion and (**B**) the mosquito oviposition pheromone. Pentane and hexane were used as the control in A and B, respectively. A binary logistic regression was used for statistical analysis. Lowercase letters indicate significant differences by likelihood ratio test in pairwise comparisons, *P* < 0.05. Ten replicates of five mosquitoes each were used in each behavioural experiment. Error bars denote the standard error of the mean (*N* = 10)

**Fig. 4:**
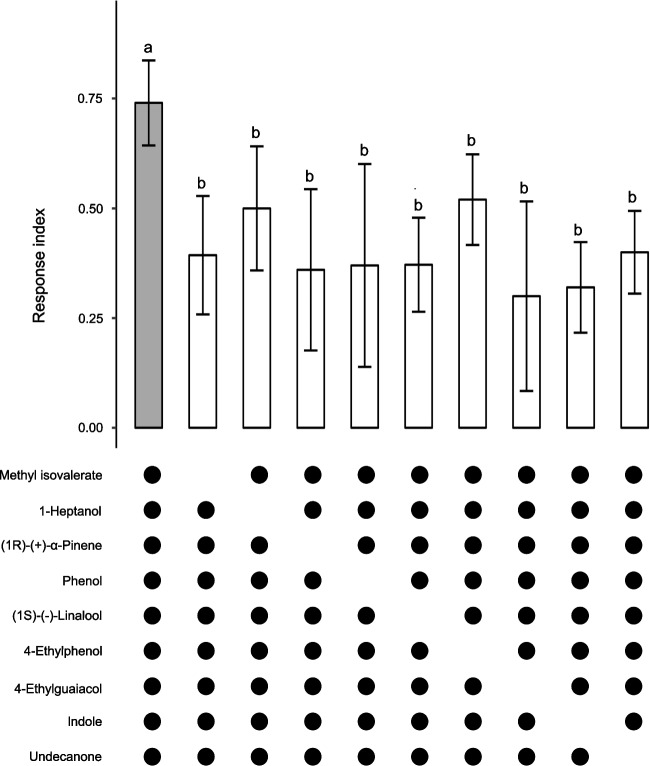
Attraction of gravid *Culex quinquefasciatus* to subtractive blends of volatile organic compounds identified in the headspace of alfalfa infusions. Attraction to the subtractive blends was significantly reduced compared to that of the full synthetic blend, as determined by binary logistic regression. Different lowercase letters indicate significant differences by odd ratios (likelihood ratio test) pairwise comparisons. Ten replicates of five mosquitoes each were used in each behavioural experiment. Error bars denote the standard error of the mean (*N* = 10)

A synthetic alfalfa infusion odour blend was constructed, based on the detected ratio of bioactive VOCs (Fig. 2), and then tested for its ability to attract gravid *Cx. quinquefasciatus* in a straight tube olfactometer*.* The blend elicited a dose-dependent attraction, in the ng min^−1^ range, which was significantly higher than that to the solvent control (pentane) (*χ*^*2*^ = 7.33, 95% CI 1.09–1.91; *P* = 0.0001; Fig. 3a). Behavioural assays, using subtractive blends, from which individual VOCs were removed from the full synthetic alfalfa infusion odour blend, unequivocally demonstrated that the complete synthetic blend is required to elicit a significant attraction of *Cx. quinquefasciatus* (*χ*^*2*^ = 8.49, 95% CI 1.53–2.47; *P* = 0.0001; Fig. 4). To evaluate the effectiveness of the synthetic alfalfa infusion odour blend, experiments were conducted to compare the behavioural responses of gravid *Cx. quinquefasciatus* to MOP. MOP elicited a dose-dependent attraction, in the µg range, which was significantly higher than that to the control, at the higher doses tested (*χ*^*2*^ = 20.92, 95% CI 3.98–4.82; *P* = 0.0001; Fig. 3b).

## Discussion

Plant infusions, including alfalfa, attract and stimulate egg laying in gravid culicine mosquitoes (this study, Afify and Galizia 2015; Bentley and Day 1989; Du and Millar 1999a; Girard et al. 2021; Isoei and Millar 1996; Khan et al. 2022; Lewis et al. 1974; McPhatter and Dubboun 2009; O’Gower 1963; Reisen and Meyer 1990; Reiter and Colon 1991). Through combined GC-EAD and GC–MS analyses, bioactive VOCs from the headspace of alfalfa infusions were identified. While indolic and phenolic compounds were the most abundant VOCs, straight-tube olfactometer assays demonstrated that these VOCs act in combination with other VOCs to elicit attraction of gravid *Cx. quinquefasciatus*. In comparison with MOP, which required a relatively high dose to induce attraction, gravid *Cx. quinquefasciatus* responded to lower doses of the synthetic alfalfa odour blend, suggesting that this blend could be a cost-effective and efficient alternative for monitoring and control of *Cx. quinquefasciatus*, in combination with other integrated vector management strategies. Further analysis is, however, required to assess the release rates of the two attractants to confirm their respective bioactivity.

Indolic and phenolic compounds are the chemical classes typically associated with decaying vegetation (Afify and Galizia 2015; Allan and Kline 1995; Du and Millar 1999a; Eneh et al. 2016; Khan et al. 2022; Millar et al. 1992). Indole, phenol, 4-ethylphenol and 4-ethylguaiacol, identified in the headspace of alfalfa infusions, have all previously been demonstrated to be associated with various plant infusions (Afify and Galizia 2015; Allan and Kline 1995; Du and Millar 1999a; Eneh et al. 2016; Khan et al. 2022; Millar et al. 1992; Navarro-Silva et al. 2009). Evaluated, either alone or in combination, these indolic and phenolic compounds are known to regulate the behavioural response of gravid culicine mosquitoes to suitable oviposition sites (Allan and Kline 1995; Beehler et al. 1994; Du and Millar 1999a; Khan et al. 2022; Millar et al. 1992). While required for the attraction of gravid *Cx. quinquefasciatus*, these VOCs act in combination with other VOCs from various chemical classes, including monoterpenes, which have not been previously described to be associated with plant infusion VOC emanates (Afify and Galizia 2015; Allan and Kline 1995; Day 2016; Du and Millar 1999a; Eneh et al. 2016; Khan et al. 2022; Millar et al. 1992). The findings of this study are in line with the recent advancements within the field of chemical ecology, which have highlighted the importance of blend recognition and discrimination in regulating odour-mediated responses of mosquitoes to various resources (Afify and Galizia 2015; Hurem and Dudding 2015; Ignell and Hill 2020; Khan et al. 2022; Wooding et al. 2020).

The MOP (Laurence and Pickett 1982, 1985) is currently the only commercially available semiochemical used for the surveillance and control of *Culex* mosquitoes (Gowrishankar and Latha 2018; Mboera et al. 2000a, b; Mihou and Michaelakis 2010; Mwingira et al. 2020; Olagbemiro et al. 2004; Otieno et al. 1988; Wooding et al. 2020). Initially produced through expensive asymmetric syntheses and routes to obtain the required racemic product (Dawson et al. 1989; Hurem and Dudding 2015; Mihou and Michaelakis 2010; Olagbemiro et al. 1999; Olagbemiro et al. 2004), MOP is currently produced through green chemistry (Pickett and Woodcock 2007), although availability and cost are still restrictive. While the results from the current laboratory assays suggest that a synthetic alfalfa infusion odour blend may be sufficient in luring gravid *Culex* mosquitoes to traps, future laboratory and field studies are required to assess possible synergies between these semiochemicals, as suggested by previous studies assessing the effect of combinations of MOP and various plant infusions (Barbosa et al. 2007; Braks et al. 2007; Blackwell et al. 1993; Day 2016; Mboera et al. 1999, 2000a, b; Michaelakis et al. 2009; Mihou and Michaelakis 2010; Mwingira et al. 2020; Olagbemiro et al. 2004; Otieno et al. 1988; Wooding et al. 2020).

The synthetic alfalfa infusion odour provides a novel lure for the surveillance and control of gravid *Culex quinquefasciatus*. A synthetic odour will overcome the inherent problems with using plant infusions, and would allow for a standard lure that will maintain its efficacy over time and in between batches. Future assessment under field conditions will allow for a broader evaluation of its efficiency in attracting other mosquito species that are attracted and prefer to lay eggs in and around fermented vegetation. Such field studies will also allow for the assessment of costs to produce long-lasting lures for professionals and consumers alike.

## Data Availability

Data is provided within the manuscript
